# Cervical cancer knowledge, attitudes, beliefs and practices of women aged at least 25 years in Harare, Zimbabwe

**DOI:** 10.1186/s12905-019-0790-6

**Published:** 2019-07-08

**Authors:** O. Tapera, G. Dreyer, W. Kadzatsa, A. M. Nyakabau, B. Stray-Pedersen, Hendricks SJH

**Affiliations:** 1 School of Health Systems and Public Health, University of Pretoria, Pretoria, South Africa; 2 Gynaecologic Oncology, Department of Obstetrics and Gynaecology, University of Pretoria, Pretoria, South Africa; 3 Radiotherapy Centre, Parirenyatwa Group of Hospitals, Harare, Zimbabwe; 4 Institute of Clinical Medicine, University in Oslo and Womens’ Clinic, Oslo University, Oslo, Norway; 5 Faculty of Dentistry and Oral Health Hospital, Sefako Makgatho Health Sciences University, Pretoria, South Africa; 6 Faculty of Health Sciences, University of Fort Hare, East London, South Africa

**Keywords:** Cervical cancer, Attitudes, Beliefs, Practices, Knowledge, Prevention, Risk factors, Harare, Treatment, Sequential explanatory mixed methods

## Abstract

**Background:**

Cervical cancer is the most common cancer and a major cause of morbidity and mortality among women in Zimbabwe yet it is preventable, early detectable and highly curable. The objective of this study was to investigate knowledge, attitudes, beliefs and practices towards cervical cancer, its prevention and treatment in Harare, Zimbabwe.

**Methods:**

Sequential explanatory mixed methods approach consisting of analytical cross sectional survey and a qualitative inquiry was used. Study population consisted of women with cervical cancer, health workers and other stakeholders who are involved in cancer control programmes. Patient survey data were collected using validated structured questionnaire in *Surveytogo* software in an android tablet. Qualitative study used key informant interviews to understand survey findings better. Data analyses for the survey involved univariate and multivariate analyses using *STATA* version 14. For qualitative study, themes in transcripts were coded and analyzed using *Dedoose* software to generate evidence for the study.

**Results:**

Participants reported different levels of knowledge of causes (23%), risk factors (71%), prevention (72%), screening (73%) and treatment (80%) of cervical cancer. Knowledge of causes of cervical cancer were negatively associated with: being aged 45 or more years (OR = 0.02; *p* = 0.004), having no household income (OR = 0.02;*p* = 0.007), household income <US$600 per month (OR = 0.02; *p* = 0.015), middle class wealth (OR = 0.01;*p* = 0.032), watching TV daily (OR = 0.01;p = 0.007) and 1–6 times per week (OR = 0.02; *p* = 0.045). Knowledge of causes of cervical cancer were also positively associated with listening to radio daily (OR = 394, CI: 11.02–1406) (*p* = 0.001) and 1–6 times a week (OR = 100, CI: 2.95–3364) (*p* = 0.010). Knowledge of prevention was only positively associated with listening to the radio daily (OR = 77, CI: 1.89–3114) (*p* = 0.022) and 1–6 times a week (OR = 174, CI: 2.42–1255) (*p* = 0.018). Major drivers of lack of knowledge for cervical cancer were: limited awareness programmes, lack of knowledge among health workers, donor prioritization of infectious diseases, infancy of cervical cancer interventions, negative attitudes towards cervical cancer and misconceptions.

**Conclusions:**

This study revealed that knowledge of causes and prevention of cervical cancer was associated with frequent radio listenership. Strengthening of health education through the packaging of messages targeting the wider society using different delivery channels is thus recommended.

**Electronic supplementary material:**

The online version of this article (10.1186/s12905-019-0790-6) contains supplementary material, which is available to authorized users.

## Background

Cervical cancer, the fourth most common cancer worldwide [1] with 80% of the cases occurring in low-income countries is a global concern [2]. Paradoxically, cervical cancer is a potentially preventable disease, yet it affects millions of women across the world [2]. In Zimbabwe, at least 2270 new cases of cervical cancer are diagnosed yearly and approximately 1451 deaths attributable to the disease are recorded annually [3]. Recent data has shown that about 79% of people are aware of cervical cancer [4] however; there is limited specific knowledge of its causes, risk factors, prevention and treatment.

In Zimbabwe, previous researchers reported that cervical cancer knowledge and understanding was poor despite the high morbidity and mortality of the disease. This lack of knowledge coupled with misconceptions about the disease and its treatment leads to poor health seeking behaviours [4]. Researchers in Uganda revealed that knowledge about cervical cancer prevention was relatively high and attitudes on screening encouraging. However, specific knowledge on the disease and its causes and screening were low [5]. In a Tanzanian study, less than 50% of nurses studied had adequate knowledge about cervical cancer and there was significant association between their knowledge levels of causes, HPV transmission and age amongst them [6]. Ahmed et al. [7] reported that market women had a fair knowledge of cervical cancer and its screening; however, there was poor knowledge of risk factors. India has the highest burden of cervical cancer in world and researchers have reported poor awareness of cervical cancer and its prevention [8]. Knowledge deficit was associated with non-adherence to cervical cancer screening amongst nurses in a Japanese study [9].

Some researchers reported levels of education and economic status as determinants of knowledge of causes, risk factors, prevention and treatment of cervical cancer [10, 11–14]. A recent study conducted in Nigeria reported that major reasons for screening using Pap smear were health worker recommendations and fear of contracting the disease. Reasons for not taking the screening intervention were lack of awareness and non-recommendation by health workers. Prior counseling by health workers and knowing someone who had cervical cancer increased knowledge and uptake of Pap smear test. However, level of education influenced knowledge of cervical cancer but not uptake of Pap smear [15]. Visanuyothin and colleagues in their study in Thailand reported that perceived barriers and knowledge of the disease and its treatment were predictors of cervical cancer screening adherence after adjusting for occupation, marital status, number of children and health insurance status in the model [16]. Researchers in Zimbabwe recently reported high knowledge of factors that caused cervical cancer amongst women from traditional churches, positive attitudes towards Pap smear test but low screening rates [1]. In 2015, the Zimbabwe Demographic and Health Survey reported that women who ever screened for cervical cancer in Harare were only 24% [17]. This study sought to bridge the information gap on the factors associated with knowledge of cervical cancer causes and prevention in the country.

## Methods

### Study design

A sequential explanatory mixed methods design was used to generate evidence for this study. This design comprise of both quantitative and qualitative methods, with the quantitative study being the major study. Qualitative inquiry was conducted to aid deeper understanding of issues, explain surprising and unexpected results from the quantitative surveys [18]. A health facility based survey was conducted between January and April 2018 among women with cervical cancer. The second phase of the study consisted of a qualitative inquiry with key informants. These participants included health workers directly involved in cervical cancer control, WHO officials, Ministry of Health policy makers and NGO programme managers. Key informants were selected using snowball sampling technique [18, 19].

### Target population

The study population consisted of women with cervical cancer, health workers and other stakeholders who are involved in cervical cancer interventions in Zimbabwe.

### Sample sizes

In the health facility based survey, 134 women with histologically confirmed cervical cancer were selected in a census at Island Hospice, Harare and Parirenyatwa Hospitals between January and April, 2018. This sample size was based on the follow of patients in these health facilities based on data from the previous year, 2017. A minimum of sample size of 80 had been determined before the study based on 2017 data; however 134 were ultimately enrolled to improve on precision of estimates. In the qualitative study the sample sizes were guided by the saturation principle [20]. The final sample size achieved for key informant interviews was 17 participants.

### Study settings

The health facility study sites were: Parirenyatwa Hospital, Harare Hospital and Island Hospice. The treating health facilities serve people from across the country; however, most of the patients recruited in this study were resident in Harare communities. All participants in the survey were aged at least 25 years (and no maximum age limit) and consented in writing before being enrolled in the study. Some of the key informants for qualitative study were selected from the same study sites as the survey. They were selected based on special characteristics such as knowledge of cervical cancer and unique experiences in the treatment of cervical cancer.

### Data collection methods

For the survey, a validated structured questionnaire [21] was administered by the researchers. The questionnaires had been programmed in *SurveytoGo* software (*Dooblo*, Israel) in android tablets. Electronic data collection allowed real time uploading of data, better quality control and reduced the time of data processing as there was no need for manual data entry. For the qualitative study, key informant interview guide (Additional file 1) was used for data collection. This study being a sequential mixed methods design, the qualitative tool was designed based on the outputs from the quantitative study analysis. The purpose of the qualitative study was to explain surprising results, outliers and put stories to data or associations in the survey.

### Quantitative analysis

Data analysis for the survey was conducted using univariate and multivariate methods. Univariate analyses were conducted to obtain proportions with respect to knowledge of causes, risk factors, prevention and treatment amongst participants. Since Likert scales were used on some variables, the responses were compressed to generate binary variables “Yes” and “No” for the univariate and bivariate analyses. The multivariate analysis outcome variables were also converted into binary variables as criteria for conducting logistic regression analysis. Logistic regression analysis was conducted to identify factors that were associated with correct knowledge of causes, prevention and treatment. The confounding variables that were adjusted for in the multivariate analysis were: sources of cervical cancer information and head of household’s occupation, education, radio listenership, watching TV, reading newspapers and accessing internet for general information. The identification of these confounders was based on literature from similar studies. All statistical analyses were conducted using *STATA* version 14 (*StataCorp*, Texas).

### Qualitative analysis

A total of 17 key informants were enrolled for the qualitative research. Participants were selected based on specific characteristics relevant to the research question and to ensure diversity of perspectives to strengthen the study outcomes.

All audio-recorded key informant interviews were transcribed verbatim. The transcripts were coded manually line by line by the researchers using *Dedoose software* (SocioCultural Research Consultants, Los Angeles) after creation of codes based on the research questions and quantitative findings. Manually generated thematic codes were processed in the same software to produce final outputs. Findings from survey and qualitative study were integrated at interpretation stage in which case qualitative findings assisted in explaining and interpreting results from the survey.

## Results

### Quantitative findings

A total of 143 participants responded to the survey these were women with cervical cancer. The mean age of participants enrolled in the survey was 52 years (SD = 12.2).

Table 1 show that women with cervical cancer had poor knowledge with respect to the causes of the disease. Only 23% of the respondents specifically reported HPV as the cause of cervical cancer. Seventy-three percent (73%) of participants had knowledge of at least one risk factor of cervical cancer. Cervical cancer patients who had knowledge about the availability of free screening services at local hospital or health facility and HIV testing being optional during screening were 73%. Eighty percent (80%) of women with cervical cancer knew about treatment modalities about the disease.

**Table 1:** Knowledge of cervical cancer causes, risk factors, prevention, screening and treatment among women with cervical cancer in Harare

Question/variable	Women with cervical cancer [*N* = 134]) (%) Correct answer
Causes	
HPV as the cause of cervical cancer	31 (23)
Prevention	
Correct prevention methods (early screening and treatment, male circumcision, eating healthy, use of condoms, sticking to one sexual partner, and abstinence from sex)	95 (71)
Risk factors	
HIV/AIDS	10 (7)
STIs	50 (37)
Multiple sexual partners without protection	22 (16)
Uncircumcised partner	7 (5)
Poor personal hygiene	1 (1)
Use of herbs or traditional medicines in vagina	8 (6)
Screening	
Hospitals/clinics in my community offer screening for cervical cancer for free	98 (73)
HIV testing is optional when being screened for cervical cancer.	98 (73)
Treatment of cervical cancer	
Treatment modalities for cervical cancer (surgery, radiotherapy and chemotherapy)	107 (80)

In Table 2, the majority (98%) of women reported that screening was important for early treatment of cervical cancer. Only 17 and 2% of women had negative attitudes that cervical cancer treatment was for people with money and the treatment was embarrassing respectively. With regards to beliefs, the majority of women had positive beliefs, however; 52 and 33% believed that cervical cancer treatment abroad was better and health workers abroad provided better treatment and care respectively. In addition, 29% of participants believed that patients treated abroad had better survival outcomes compared to those treated locally. Women who had access to a regular doctor were relatively low, 48%. However, participants who reported ever screening for cervical cancer in their lifetime were 95%. Proportion of participants who reported screening at least once in their lifetime was relatively low, 66%.

**Table 2:** Attitudes, beliefs and practices towards cervical cancer, its screening and treatment among women with cervical cancer in Harare

Questions	Women with cervical cancer [*N* = 134] (%) Yes
Attitudes	
Cervical cancer treatment is for people with money	23 (17)
Cervical cancer treatment procedure is embarrassing	1 (2)
Screening is important for early treatment of cervical cancer	131 (98)
Beliefs	
Cervical cancer treatment saves lives	133 (99)
Cervical cancer treatment gives a woman and their family peace of mind	133 (99)
Cervical cancer treatment gives a woman control over her health.	133 (99)
Cervical cancer treatment is not painful	70 (28)
Cervical cancer treatment has no side-effects.	83 (69)
Cervical cancer treatment is for all women regardless of background	128 (95)
Cervical cancer cannot be treated	11 (8)
Cervical cancer patients do not survive long even when treated	7 (5)
Cervical cancer is best treated with herbs/traditional medicines	14 (10)
Cervical cancer is best treated using spiritual means performed by prophets and pastors.	14 (10)
Cervical cancer treatment is best done abroad	73 (54)
Health professionals abroad provide better care for cervical cancer patients.	45 (33)
Cervical cancer patients treated abroad have better survival chances	39 (29)
Practices	
Do you have a regular doctor whom you see when you require health services?	64 (48)
Women ever screened for cervical cancer	127 (95)
Women screened at least once in their life time	88 (66)

Table 3 shows that knowledge of causes of cervical cancer was negatively associated with being aged 45 years or more (*p* = 0.004), having no household income (*p* = 0.007), household income less than US$600 per month (*p* = 0.015), having middle class wealth (*p* = 0.032), watching TV daily (*p* = 0.007) and between once and 6 times per week (*p* = 0.045). Factors positively associated with correct knowledge of causes were listening to the radio daily (*p* = 0.001) and between once and 6 times per week (*p* = 0.010). Factors positively associated with cervical cancer prevention knowledge were listening to the radio daily (*p* = 0.022) and between once and 6 times per week (*p* = 0.018).

**Table 3:** Factors associated with correct knowledge of causes and prevention of cervical cancer among 134 women with the disease in Harare

	Women with cervical cancer [*N* = 134]			
	Correct knowledge of causes		Correct knowledge of prevention	
Variables	OR, (95% CI)	*p* value	OR, (95% CI)	*p* value
Residence				
Urban	25.12 (0.82 to 767)	0.065	0.38 (0.01 to 19.1)	0.625
Rural	Ref	–	Ref	–
Age (years)				
25–44	Ref	–	Ref	–
45 or more	0.02 (0.00 to 0.15)	**0.004**	0.17 (0.01 to 5.63)	0.328
Marital status				
Married/co-habiting	6.00 (0.08 to 473)	0.599	0.08 (0.00 to 72.0)	0.716
Never married	–	–	–	–
Widowed	0.99 (0.07 to 14.10)	0.994	0.04 (0.00 to 1.54)	0.085
Divorced or separated	Ref	–	Ref	–
Religion				
Roman Catholic	1.80 (0.03 to 120)	0.787	0.35 (0.00 to 42.0)	0.665
Protestant	2.27 (0.04 to 143)	0.697	58.64 (0.32 to 1069)	0.125
Pentecostal	0.56 (0.00 to 39)	0.786	0.07 (0.00 to 5.56)	0.238
Apostolic sect	5.85 (0.05 to 709)	0.470	169 (0.36 to 7965)	0.102
Other	Ref	–	Ref	–
Occupation				
Unemployed	2.87 (0.13 to 65)	0.508	1.19 (0.05 to 26.6)	0.912
Professional	0.00 (0.00 to 6.84)	0.116	284 (0.00 to 7340)	0.567
Other	Ref	–	–	**–**
Household monthly income (US$)				
No income	0.02 (0.00 to 0.07)	**0.007**	0.13 (0.00 to 74.0)	0.843
< 600	0.02 (0.00 to 0.13)	**0.015**	0.03 (0.00 to 1.02)	0.717
600–1000	0.52(0.00 to 107)	0.897	4.12 (0.00 to 708)	0.200
1200 or more	Ref	–	Ref	**–**
Wealth quintiles				
Poor	Ref	–	–	–
Middle	0.01 (0.00 to 0.66)	**0.032**	–	–
Rich	0.33 (0.00 to 3.73)	0.157	–	–
Watching TV per week				
Daily	0.01 (0.00 to 0.14)	**0.007**	0.18 (0.00 to 36.1)	0.525
1–6 times	0.02 (0.00 to 0.92)	**0.045**	1.67 (0.04 to 62.9)	0.782
Never	Ref	–	–	**–**
Listening to radio per week				
Daily	394 (11.02 to 1406)	**0.001**	77 (1.89 to 3114)	**0.022**
1–6 times	100 (2.95 to 3364)	**0.010**	174 (2.42 to 1255)	**0.018**
Never	Ref	–	Ref	–
Reading newspaper per week				
Daily	14.5 (0.64 to 3263)	0.333	188 (0.36 to 9840)	0.076
1–6 times	1.60 (0.10 to 243)	0.857	0.04 (0.00 to 2.68)	0.097
Never	Ref	–	10.1 (0.01 to 7837)	0.418

Bold shows factors that were significant

### Qualitative findings

#### Theme 1: drivers of limited knowledge about cervical cancer causes, risk factors, prevention and treatment

Surveys suggested limited specific knowledge about cervical cancer in communities and among patients. Drivers of lack of knowledge that emerged in the qualitative study included: limited awareness programmes in communities, infancy of cervical cancer awareness programmes, donor priorities to infectious diseases (vertical interventions), lack of knowledge amongst health workers, negative attitudes towards cervical cancer and misconceptions. The following quotes show some of the drivers of lack of knowledge reported:

“*You walk into a rural health facility and you ask nurses about cervical cancer or cancer in general but they have no clue of what it is but we are asking people in the community to go to the clinic where the nurses don’t know anything about cancer*”- Cancer Centre Midwife, key informant.

*“We also talked of education I don’t think we are reaching enough in terms of education because we just give health education to those that would have visited the health centre and we don’t have outreach programmes that help us reach the communities”,*-Harare Hospital VIAC nurse, key informant

*“………the minute you get into the realm of cancer there is fear of the unknown…..*so people are *in a comfort zone where they don’t want to delve”* -Harare Pathologist, key informant.

#### Theme 2: drivers of low utilization of screening services

Key informant interviews pointed out some of deterring factors resulting in this low usage of early screening leading to late disease presentations. Some of the factors reported by most respondents were: lack of knowledge, belief that cervical cancer was not treatable, misconception that cervical cancer was caused by witchcraft or avenging spirits, fear of the unknown, limited screening services, health worker negative attitudes, lack of funds for transport, negative attitudes towards screening and social norms such as a person should only use the health facility when they are sick or in pain and the need for husband or partner approval or financial support to visit the health facility to be screened. Some of the respondents had this to say:

*“It is fear of the unknown and lack of knowledge, those are the two things that are really hampering the campaign against cancer of the cervix*”- Harare Pathologist, key informant

*“I think there is very little awareness of the population about cervical cancer. I think it’s something which was not actively talked about to the community as much was HIV or TB*”.-Harare Senior Gynaecologist, key informant

## Discussion

Despite relatively high knowledge of risk factors, prevention and treatment, knowledge of cause remains suboptimal. Women with cervical cancer had low knowledge of causes of the disease with only 23% reporting HPV as the cause. Seventy-three percent (73%) of participants had knowledge of at least one risk factor of cervical cancer. Cervical cancer patients had better knowledge about the availability of free screening services at local hospital or health facility and HIV testing being optional during screening. Eighty percent (80%) of women with cervical cancer knew about treatment modalities of the disease. Majority (98%) of women reported that screening was important for early treatment of cervical cancer. Only a few participants reported negative attitudes towards treatment of cervical cancer. With regards to beliefs, the majority of women had positive beliefs, however; 54 and 33% believed that cervical cancer treatment abroad was better and health workers abroad provided better treatment and care respectively. Only 29% of participants believed that patients treated abroad had better survival outcomes compared to those treated locally. Women who had access to a regular doctor were relatively low, 48% while 95% of participants reported ever screening for cervical cancer in their lifetime. A relative low number of women with cervical cancer (66%) reported screening at least once in their lifetime. Knowledge of causes of cervical cancer was negatively associated with being aged 45 years or more (*p* = 0.004), having no income (*p* = 0.007), income less than US$600 per month (*p* = 0.015), being in the middle class wealth quintile (*p* = 0.032), watching TV daily (*p* = 0.007) and between once and 6 times per week (*p* = 0.045). Factors positively associated with correct knowledge of causes were listening to the radio daily (*p* = 0.001) and between once and 6 times per week (*p* = 0.010). Factors positively associated with cervical cancer prevention knowledge were listening to the radio daily (*p* = 0.022) and between once and 6 times per week (*p* = 0.018).

### Causes

Knowledge about the causes of cervical cancer was low among cervical cancer patients. This finding suggests that there is still limited awareness or health education with regards to the basic information on cervical cancer. The assumption would be that cervical cancer patients because of their condition would tend to be exposed more to information or have positive attitude towards information about their condition. However, findings suggest that this may not be so. About 72% of women with cervical cancer were able to report at least one risk factor of cervical cancer (HPV, HIV/AIDS, STI, poor personal hygiene, multiple sex partners without protection, sex with uncircumcised partner, and use of herbs in the vagina). However, HPV as a cause of cervical cancer was only reported by a small proportion of participants, 23%, confirming that specific knowledge about the cause of this condition is still limited in Zimbabwe. Our findings suggest relatively high knowledge of risk factors of cervical cancer as compared to other studies. In an Ethiopian study, only 42% of participants reported at least one risk factor of cervical cancer [10]. Researchers in Nigeria reported knowledge of risk factors as low as 15.6% in a recent study [15]. In Tanzania, multiple sexual partners and history of HPV infection as risk factors for developing cervical cancer were reported by 47.4 and 43.1% of respondents respectively [6].

Knowledge of causes of cervical cancer reported was associated negatively associated with being aged 45 years or more (*p* = 0.004), having no income (*p* = 0.007), income less than US$600 per month (*p* = 0.015), being in the middle class wealth quintile (*p* = 0.032), watching TV daily (*p* = 0.007) and between once and 6 times per week (*p* = 0.045). However, listening to the radio frequently was positively associated with knowledge of causes of cervical cancer. These results suggest that radio messaging may have been effective in influencing cervical cancer awareness. The findings may also suggest that awareness or health education programmes may be biased towards radio programmes or that the packaging of messages is appropriate for radio audience. Programmes in television may not be properly packaged to influence knowledge of causes but may be reinforcing fear, stigma, myths and misconceptions in the population. However, this was not explicitly explored in our qualitative study and future studies should consider understanding the role of television in knowledge of causes and prevention of cervical cancer. Our findings support those of Mitiku & Tefera [10] who found that better income was associated with knowledge of cervical cancer. A Nigerian study reported that being younger than 30 years was associated with lack of knowledge of cervical cancer or its screening [22], and this is in contrast to our results which revealed that being 45 or older was associated with lack of knowledge. Our results did not find any association of knowledge of causes with levels of education and this directly contrast those of a similar study in Zimbabwe, which suggested that knowledge of cervical cancer was associated with having at least secondary education [23].

### Prevention

There was suboptimal knowledge of cervical cancer prevention reported among women with cervical cancer in our study, 71%. This suggests that lack of prevention knowledge for cervical cancer may be a possible risk factor for disease incidence [24, 25]. Mukama et al. [5], in Uganda reported a relatively lower knowledge of preventive measures (62%). The factor associated with preventive knowledge was listening to the radio frequently. These results also suggest that radio programmes may be a better platform for disseminating cervical cancer awareness and education. Packaging of messages for cervical cancer could be targeting radio audience. These findings contrasts those of Ayinde [22] who reported that married women were more likely to be aware of cervical cancer compared to single women. Our study contrast the findings from a Ugandan study which revealed that living in peri-urban and urban areas, having a higher monthly income and having had an HIV test were associated with knowledge about cervical cancer prevention [5].

### Treatment

Our study revealed that knowledge of treatment was relatively high among women with cervical cancer (80%). This high knowledge could be explained by frequent interface with health facilities and perhaps desperation for treatment for a disease commonly believed to be untreatable [26–29]. It is imperative that awareness or health education be biased towards risk factors and causes to reduce the risk of developing cervical cancer which is costly to treat and is a significant threat to life [30]. As revealed in the qualitative phase of this study, there are still some gaps in the packaging of awareness or health education interventions for cervical cancer. In our setting, cervical cancer treatment is marred with myths and misconceptions though only a small proportion (8%) of the respondents did not believe that the disease was treatable. To our knowledge, this study is the first to report knowledge levels with regards to treatment of cervical cancer in our context. There is generally little information about the knowledge of treatment in developing countries with most studies focusing on primary and secondary interventions for cervical cancer.

### Attitudes

Generally, women participants in our study had positive attitudes towards cervical cancer, its screening and treatment. The majority agreed that early screening was important for early treatment however; a few women reported that cervical cancer treatment was for people with money and treatment procedures were embarrassing. These negative attitudes have an influence on the uptake of treatment services [5]. Low screening uptake and poor health seeking behaviours may be explained by the negative attitudes towards treatment despite positive attitudes towards screening itself [5, 11–14]. Our qualitative findings also reported negative attitudes as a barrier to both preventive and curative interventions for cervical cancer.

### Beliefs

Cervical cancer treatment is marred with many beliefs, myths and misconceptions [31]. Most participants had positive beliefs with regards to cervical cancer, its screening and treatment. However, a relatively high proportion believed that treatment, care of health workers abroad and survival outcomes for those treated abroad were better. These findings suggest relatively low levels of confidence in local health services or local capacity for cervical cancer treatment. This belief could also be emanating from numerous newspaper and online reports of affluent members of society including senior government officials and politicians who seek and receive treatment of chronic diseases abroad. Medical tourism has been reported to have a negative effect on the perceptions and development of local health service delivery [32].

### Practices

Our study showed that the majority of women with cervical cancer had used screening services in their lifetime. However, they had limited access to regular doctors for health services and this could be a predisposing factor to late presentation or limited access to treatment. All the women enrolled in the study had at least locally advanced disease (>stage 1B2). These findings suggest the use of screening services for “diagnosis” among women with overt symptoms of cervical cancer. Despite the relatively high levels of awareness of cervical cancer, its prevention and treatment, our study suggests suboptimal routine uptake of screening for cervical cancer among asymptomatic women in Harare. These results are in tandem with ZDHS (2015) findings which reported that only 24% of healthy women had ever screened for cervical cancer in their lifetime [17]. These results are unexpected given the relatively high levels of knowledge of cervical cancer, prevention and treatment in our context. Our qualitative study explained some of the reasons of low screening uptake among them: lack of knowledge, beliefs that cervical cancer was not treatable, misconception that cervical cancer was caused by witchcraft or avenging spirits, fear of the unknown, limited screening services and negative health worker attitudes. Lack of funds for transport and negative attitudes towards screening and poor health seeking behavior with a common attitude that a person should only use health facilities when they are sick or in pain could be contributing to low cervical cancer screening uptake in our settings. Social norms that necessitate women to seek husband or partner approval or financial support to get screened may also explain low uptake of screening services. Considering other low-income settings, in Nigeria uptake of screening services was reported to be as low as 23% and this was not surprising given the low levels of awareness of cervical cancer [15]. Again in Tanzania, a study amongst nurses revealed low screening uptake, only 15%, using the Pap smear technique [6]. Surprisingly in a similar study in Japan, a high-income country, only 14% of nurses had screened for cervical cancer in the previous 2 years [9]. In contrast in Thailand, Visanuyothin and colleagues reported a high Pap smear uptake of 77% amongst women in a recent study [16].

This study encountered some limitations which would deserve to be accounted for in interpreting the findings. Firstly, this study was conducted in Harare, the capital city of Zimbabwe where cervical cancer awareness, screening and treatment services are largely available compared to other parts of the country. Therefore, the results of this study may not be generalizable to other contexts and a follow-up national study would be imperative in future. Secondly, the study was conducted at a time health workers had series of strikes and therefore there may have been selection bias in the characteristics of patients who were scheduled for reviews or treatments particularly at Parirenyatwa and Harare Hospitals. This study comprised of a cross sectional survey whose outcomes cannot be used to infer causal relationships. Our qualitative study could not explore the underlying issues behind watching TV being negatively associated with knowledge of causes and prevention and further studies are recommended. However, this study had its own strengths; firstly this study used mixed methods with a diverse study population to understand the subject under investigation better. Most studies cited have used either qualitative or quantitative methods and for policy recommendations mixed methods provide better outcomes [18, 33]. Secondly, this study, to the best of our knowledge, is the first in Zimbabwe to comprehensively investigate knowledge, attitudes, beliefs and practices about cervical cancer, its screening and treatment. Many previous studies focused on primary and secondary prevention of cervical cancer but there is a general paucity of information on tertiary interventions in the context of low-income settings.

## Conclusions

This study revealed that knowledge of causes and prevention of cervical cancer was associated with frequent radio listenership. Despite relatively high levels of knowledge of cervical cancer prevention, screening and treatment, specific knowledge of causes, risk factors, access to primary care and routine utilization of screening services remains suboptimal. Strengthening of health education through the packaging of messages targeting the wider society using different delivery channels such as community campaigns, mobile phone messages and health workers is thus recommended. Investment in health systems to improve universal coverage of health services is another important step in low income settings.

## Additional file

Additional file 1: Key informant interview guide (DOCX 22 kb)

## Data Availability

The datasets used and/or analyzed during the current study are available from the corresponding author on reasonable request.

## Abbreviations

AIDS: Acquired immunodeficiency syndrome
FGD: Focus group discussion
HIV: Human immunodeficiency syndrome
HPV: Human papilloma virus
NGO: Non-governmental organization
SD: Standard deviation
STI: Sexually transmitted infection
VIAC: Visual inspection with acetic acid cervicography
ZDHS: Zimbabwe demographic and health survey
