# Supplementary material for: Cervical cancer knowledge, attitudes, beliefs and practices of women aged at least 25 years in Harare, Zimbabwe
Source: BMC Womens Health. 2019 Jul 8;19:91. doi: 10.1186/s12905-019-0790-6 (PMC6615311; doi:10.1186/s12905-019-0790-6)
Supplement: Supplementary file 1 — Key informant interview guide (DOCX 22 kb) [file 12905_2019_790_MOESM1_ESM.docx]

**KEY INFORMANT INTERVIEW GUIDE**

| **DISCUSSION QUESTIONS** |
| --- |
| **Demographics of respondent**  • Can you please tell me more about yourself : Probe for: Age, ethnicity, education, occupation, place of residence, interaction with communities and how long they have been interacting with communities? |

| **Knowledge of cervical cancer**   - In your opinion do people know about cervical cancer? Probe for general knowledge, causes, signs and symptoms and risk factors. - According to your own understanding what do you think are some of the causes, signs and symptoms and risk factors of cervical cancer that you know? - Can you tell me more about people’s awareness of preventing cervical cancer in your community? - What are some of the measures that can be taken to prevent cervical cancer? Probe for screening and treatment of precancerous lesions, HPV vaccination and male circumcision of partners. - What are your opinions of your community with regards to risk of developing this disease and why do you say so? |
| --- |
| **Experiences of cervical cancer**   - What have been your experiences with cervical cancer survivors? - What are some of the reasons why women go for cervical cancer screening? Probe for routine screening, health problems, health worker advice, friend or relative advice? - What are your opinions on the awareness of treatment and palliative services for cervical cancer in your community? - Tell me more about your knowledge of cervical cancer treatment and palliative care services in Harare? Probe for where the services are offered. - Can you tell me what you know about support from partners, friends and families for cervical cancer patents in your community? - How do you think partner, friend or family support help cervical cancer patients? - What roles are churches in Harare playing to support cervical cancer patients? Probe for social, emotional and spiritual support. What are the general perceptions of people in your community with regards to cervical cancer? Probe for attitudes, beliefs, misconceptions and fears? |
| **Access to cervical cancer treatment and palliative care**   - Tell me about cervical cancer treatment and palliative services in health facilities in Harare? Probe for names of health facilities. - Can you tell me your opinions about access to treatment and palliative services in Harare? Probe for who has better access and why? - Can you tell me what you know about how cervical cancer is treated in health facilities? Probe if there are other means through which cervical cancer may be treated apart from health facilities? - Who do you think can best treat and manage cervical cancer and why do you think so? - What are your opinions about service fees for cervical cancer treatment in Harare? Probe for affordability to patients or their families? - What do you think about the availability of treatment services in Harare are adequate to cover all cervical cancer patients? Probe for reasons of response? - What do you think about the adequacy of doctors and specialists to treat cervical cancer in Harare? Probe for reasons of response? - Do you think about the training of doctors and specialists to provide good treatment services to cervical cancer patients? - What do you know about palliative care in health facilities in Harare? Probe for names of some of the facilities? - Tell me what you know/think about the adequacy of palliative services to cover the patients who need such services? - Describe some the challenges that patients in your community have experienced [or experience] in trying to access cervical cancer treatment or palliative care? |
| **Utilization of cervical cancer treatment and palliative care**   - Can you tell me more about your understanding of health seeking behaviors by women in your community for treatment and palliative services for cervical cancer in Harare? - In your opinion do think your community understands well treatment and palliative services offered for cervical cancer in health facilities? - To your knowledge, tell me more about the treatment and palliative services being accessed by women in your community? - What is your opinion of the effectiveness of treatment and palliative services provided in health facilities in Harare? - What do you think are some of the challenges that cervical cancer patients and their families are facing in using treatment and palliative care services in Harare?   **Health services**   - Tell me about the information that health facilities provide about cervical cancer to your community? - According your understanding, what are the experiences of women when they notice signs and symptoms for cervical cancer? Probe about early health seeking behaviors? - What are your opinions about adequacy of equipment, drugs, beds and other infrastructure to provide cervical treatment and palliative care in health facilities in Harare? - What are your opinions about the adequacy of health workers (nurses, nurse aids, doctors, specialists, pharmacists, radiographers and laboratory scientists) to provide treatment and palliative care of cervical cancer patients in health facilities in Harare? - Can you tell me more about your opinions on the fees that are charged for treatment services by your health facility? - To your knowledge what are the experiences of patient who cannot pay or do not afford to pay for treatment or other procedures at health facilities? - What are your perceptions on the quality of services provided at health facilities? Probe for attitudes of nurse aids, nurses, doctors, specialists, radiographers, pharmacists and other health workers? - In your opinion what are the perceptions and attitudes of your local leadership (herdmen, counsellors, and chiefs) on people using health services when they are sick or for routine checkups? - What do you think should be done at health facilities and generally in Zimbabwe to improve cervical cancer treatment and palliative care services? |

**Remarks:** Thank the participant for their time and proceed to the next one!--------------**The End------------**
